# Syngeneic B16F10 Melanoma Causes Cachexia and Impaired Skeletal Muscle Strength and Locomotor Activity in Mice

**DOI:** 10.3389/fphys.2017.00715

**Published:** 2017-09-29

**Authors:** Fabrício A. Voltarelli, Fernando T. Frajacomo, Camila de Souza Padilha, Mayra T. J. Testa, Paola S. Cella, Diogo F. Ribeiro, Donizete X. de Oliveira, Luciana C. Veronez, Gabriela S. Bisson, Felipe A. Moura, Rafael Deminice

**Affiliations:** ^1^Department of Physical Education, Faculty of Physical Education and Sport, State University of Londrina Londrina, Brazil; ^2^Department of Physical Education, Faculty of Physical Education, Federal University of Mato Grosso Cuiabá, Brazil; ^3^Program of Molecular Carcinogenesis, Brazilian National Institute of Cancer Rio de Janeiro, Brazil; ^4^Department of Maternal-Infant Nursing and Public Health, Ribeirao Preto College of Nursing, University of São Paulo São Paulo, Brazil

**Keywords:** cancer, melanoma, inflammation, muscle strength, general locomotor activity

## Abstract

Muscle wasting has been emerging as one of the principal components of cancer cachexia, leading to progressive impairment of work capacity. Despite early stages melanomas rarely promotes weight loss, the appearance of metastatic and/or solid tumor melanoma can leads to cachexia development. Here, we investigated the B16F10 tumor-induced cachexia and its contribution to muscle strength and locomotor-like activity impairment. C57BL/6 mice were subcutaneously injected with 5 × 10^4^ B16F10 melanoma cells or PBS as a Sham negative control. Tumor growth was monitored during a period of 28 days. Compared to Sham mice, tumor group depicts a loss of skeletal muscle, as well as significantly reduced muscle grip strength and epididymal fat mass. This data are in agreement with mild to severe catabolic host response promoted by elevated serum tumor necrosis factor-alpha (TNF-α), interleukin-6 (IL-6) and lactate dehydrogenase (LDH) activity. Tumor implantation has also compromised general locomotor activity and decreased exploratory behavior. Likewise, muscle loss, and elevated inflammatory interleukin were associated to muscle strength loss and locomotor activity impairment. In conclusion, our data demonstrated that subcutaneous B16F10 melanoma tumor-driven catabolic state in response to a pro-inflammatory environment that is associated with impaired skeletal muscle strength and decreased locomotor activity in tumor-bearing mice.

## Introduction

Cancer cachexia is a complex syndrome characterized by progressive weight loss, anorexia, muscle loss and weakness. Muscle wasting have been emerging as one of the principal components of cancer cachexia, leading to progressive impairment of work capacity (Fearon et al., 2011) a significant hallmark of poor prognosis in cancer patients (Christensen et al., 2014). The causal understanding of the muscle wasting in cancer cachexia is complex; the pro-inflammatory scenario however, is thought to play a prominent role (Ebrahimi et al., 2004). Elevated TNF-α, IL-6 and IL-1β are associated with weight loss, hypercatabolism, reduced food intake and muscle loss in cancer patients (De Larichaudy et al., 2012; Yuan et al., 2015). Thus, elevated inflammatory interleukins and muscle wasting during tumor development can significantly modulate muscle strength and locomotor capacity in tumor-bearing mice, some of the most critical endpoints associated with cancer morbidity and mortality (Christensen et al., 2014).

Melanoma is the deadliest form of skin cancer. When diagnosed at localized stages (I and III), the five-year survival rates correspond to 98%; however, this dramatically drops to 5–19% when metastases is presented (Sandru et al., 2014; American Cancer Society, 2017). Despite early stages melanomas are not related with weight loss, the appearance of metastatic and/or solid tumor melanoma is related to cachexia development (Kawamura et al., 1999b; Das et al., 2011). Melanoma metastasis has a simultaneous lymphatic and hematogenous spread, with the potential to metastasize in any organ (Belhocine et al., 2006). Recent data has been demonstrated lung, gastrointestinal tract, extra-regional lymph nodes, liver and prostate are the principal target of metastatic melanoma; all of than with elevated rate of cachexia (40 to 80%) and mortality (von Haehling et al., 2016).

To better unveil melanoma tumor biology and tumor-host interaction, a number of murine models have been widely used in preclinical melanoma studies (Becker et al., 2010; Kuzu et al., 2015). The most widely used cell type in the melanoma model is the B16 cell line and two subclones (F1 and F10) that spontaneously form tumors after syngeneic transplantation in C57BL/6J mice (Fidler and Nicolson, 1976). The syngeneic model involves the induction of tumor cells into the same species and genetic background (Darro et al., 2005; Bobek et al., 2010). These models have important benefits over xeno-transplantation or genetically modified models since mice possess a normal immune system that opens a useful platform for new immunotherapy and adequate host response (Becker et al., 2010). However, a few number of studies have demonstrated B16 cells induces cachexia in mice following intraperitoneal inoculation through higher lipase lipoprotein activity (Kawamura et al., 1999a,b; Das et al., 2011). Indeed, there is a paucity of information dealing with tumor-host interaction in mice carrying B16F10 melanoma cells regarding cancer cachexia, specially the interaction among cancer cachexia components and skeletal muscle strength and locomotor activity. Thus, we aimed to investigate the cachexia development response to B16F10 tumor cells inoculation and it interactions with skeletal muscle adaptations (mass and strength) and mice locomotor activity. We hypothesized muscle loss and pro-inflammatory condition induced by tumor development play a pivotal role on reduced muscle strength and decreased locomotion and exploratory activity in B16F10 tumor-bearing mice.

## Methods

### Animals

Twenty male C57BL/6 mice, 6–8 weeks old, were initially obtained from the Central Creation Unit of University of Sao Paulo (USP), Ribeirao Preto, Brazil and were used in this study. Mice were maintained under controlled temperature (24 ± 1°C), light (12 h of light/12 h of darkness), relative air humidity (60-70%) and allowed free access to water and food. After 1 week of local animal care facility adaptation, animals were randomly divided into two groups: Sham inoculated mice (*n* = 8) and tumor-bearing mice (*n* = 12). Animals were monitored three times a week for body weight, food and water intake, and tumor dimensions and euthanized 28 days after tumor cells inoculation. The daily food intake was calculated by the difference between the weight of the received food in their home cage hopper (lab balance Shimadzu BL320H) and the weight of the remained food 24 h after. The current study was approved by the Ethics Committee in Animal Experimentation of the State University of Londrina (#28336.2014.38), which follows the recommendations of the Brazilian Code for Use of Laboratory Animals (Law No. 6638, of May 8th, 1979 and Decree No. 26645 of July 10th, 1934).

### Tumor cells inoculation

B16F10 melanoma cells were cultured in RPMI 1640 medium (Gibco, Invitrogen) supplemented with 10% of fetal bovine serum (Gibco, Invitrogen), 100 μg/ml of streptomycin and 100 units/ml of penicillin at 37°C and 5% of CO_2_. For inoculation, cells were removed from culture flasks by adding 0.05% of trypsin solution, centrifuged and resuspended in sterile PBS in order to obtain a solution containing 5 × 10^4^ cells/ml. Cell viability was determined by trypan blue exclusion. Finally, C57BL/6 mice were subcutaneously injected with 5 × 10^4^ cells/animal (0.1 ml) into the right thigh. As a negative control, the sham group was inoculated with 0.1 ml PBS only. Two of initial 12 tumor-bearing animals died during the study; other two animals did not develop any apparent tumors after B16F10 inoculation and were excluded from the study. Therefore, a total of eight tumor-bearing mice were considered for the study; the same number of animals was considered for the sham group.

### Tumor assessment

Tumor growth was monitored by digital caliper (Sagyma Plus, 0–150 mm) three times a week using two-dimensional measures. Tumor volume was calculated using a standard solid tumor formula V = 1/2^*^(D^*^d2) (Goto et al., 2000); being V, volume, D higher diameter and d lower diameter. The same examiner performed all measures in order to minimize bias.

### Maximal muscle strength analysis

After 2 weeks of adaptation to protocol, the grip strength was determined using a dynamometer EEF 305 Grip Strength Meter (Insight®, Ribeirão Preto, Brazil) containing a pyramidal platform adapted to forelimbs. The test consisted of pulling the tail of the animal in order to cause their limbs to touch the grid of the equipment, which has a traction strength sensor. Three measures were performed in each mouse, with corresponding intervals of 15–12 s between sets, and with the tension administered gradually and consistently with the limitations of each animal. The quantitative data used corresponds to the mean strength of three attempts performed by the animals. The tests were carried out under the same experimental conditions.

### Locomotion and exploratory activity

Locomotion and exploratory activity was determined using an open field arena (40 × 40 cm^2^) that was divided equally into lines drawn on the chamber floor for visual scoring of the activity by the experimenter (Prut and Belzung, 2003). Each mouse was placed in the center of the field arena and was allowed to freely explore the chamber for 30 s, followed by a 5-min test length. Overall locomotor activity (measured with video track digital camera, Longitech, C920, 30 Hz fixed above 60 × 60 cm rigid box) was recorded, as well as the amount of time and distance spent and numbers of entries in the center area. During the tests, the video sequences were stored in a personal computer for analysis. Using an automatic tracking method via DVideo software interface (Figueroa et al., 2003, 2006), we obtained the trajectories of mice. The two automatic procedures of segmentation-background subtraction and tracking-generated the mouse's positions as a function of time over the entire test. Background subtraction is a very common method used for segmenting moving objects, which consists of the difference between a set of images and its background model.

Before the tests, we obtained the coordinates of four specific points relative to a coordinate system associated with the box. The corresponding projections of these points in the image were determined with DVideo software. The homography parameters of the image-object transformation were then calculated based on the DLT (Direct Linear Transformation) proposed by Abdel-Azis and Karara (1971) and mouse two-dimensional (2D) coordinates relative to the box coordinate system were obtained. Mice naturally prefer to be near a protective wall rather than be exposed to danger in an open area; however a competing foraging will motivate them to explore (Hefner et al., 2007).

### Euthanasia and preparation of tissues

The animals were euthanized using anesthetic overdose with ketamine 0.02 g and xylazine 0.004 g. Subsequently, 1 ml of blood was collected from the abdominal aorta, stored in heparinized tubes and centrifuged for plasma separation. Plasma samples were stored at −80°C for further analysis. Liver, epididymal fat pads and soleus and gastrocnemius muscles were carefully dissected and weighted. The sum of both skeletal muscles (∑) was used as muscle mass parameter. Tumors were carefully dissected from the upper-left flank for weighing, and were subsequently cut in half and stored in liquid nitrogen. The cachectic state was determined using the cachexia index that consider the body weight gain of control mice and the tumor mass in tumor-bearing mice, according to the following equation:[(Initial body weight - final body mass + (mass of the tumor) + control weight gain) x 100 / (initial body mass + mass gain control group)] (Martins et al., 2016). Cachexia index is a tool to determine weight loss related to control animals. Animals were considered cachectic when presented weight body loss >5% (Fearon et al., 2011).

### Biochemical analysis

For biochemical analysis, plasma lactate dehydrogenase (LDH; Commercial Kit Labteste/LDH Liquiform/Ref: 86-2) was measured using a plate reader (Epoch® Bio Tek, Winnoski, USA). The concentrations of plasma cytokines TNF-α (EBioscience Ref: 88-7340-88), interleukin-6 (EBioscience Ref: 88-7064-88) and interleukin-10 (EBioscience Ref: 88-7105-88) were determined using ELISA commercial available kits in a plate reader (Epoch® Bio Tek, Winnoski, USA).

### Statistical analyses

The statistical analyses were performed using IBM SPSS Software (version 21). The normality was evaluated using the Kolmogorov-Smirnov test. Normal data were analyzed by Student's *T*-test for independent samples and the results expressed as Mean ± Standard Deviation (SD). Food intake and tumor volume over time were compared using ANOVA two way differences b-test. The *Pearson* correlation coefficient test was used to determine association among skeletal muscle strength, locomotor-like activity and cachexia characteristics (body weight loss, reduced adipose tissue and skeletal muscle loss, elevated inflammatory interleukins and anorexia). In all cases, the differences were considered significant when *P* < 0.05.

## Results

**Figure 1:**
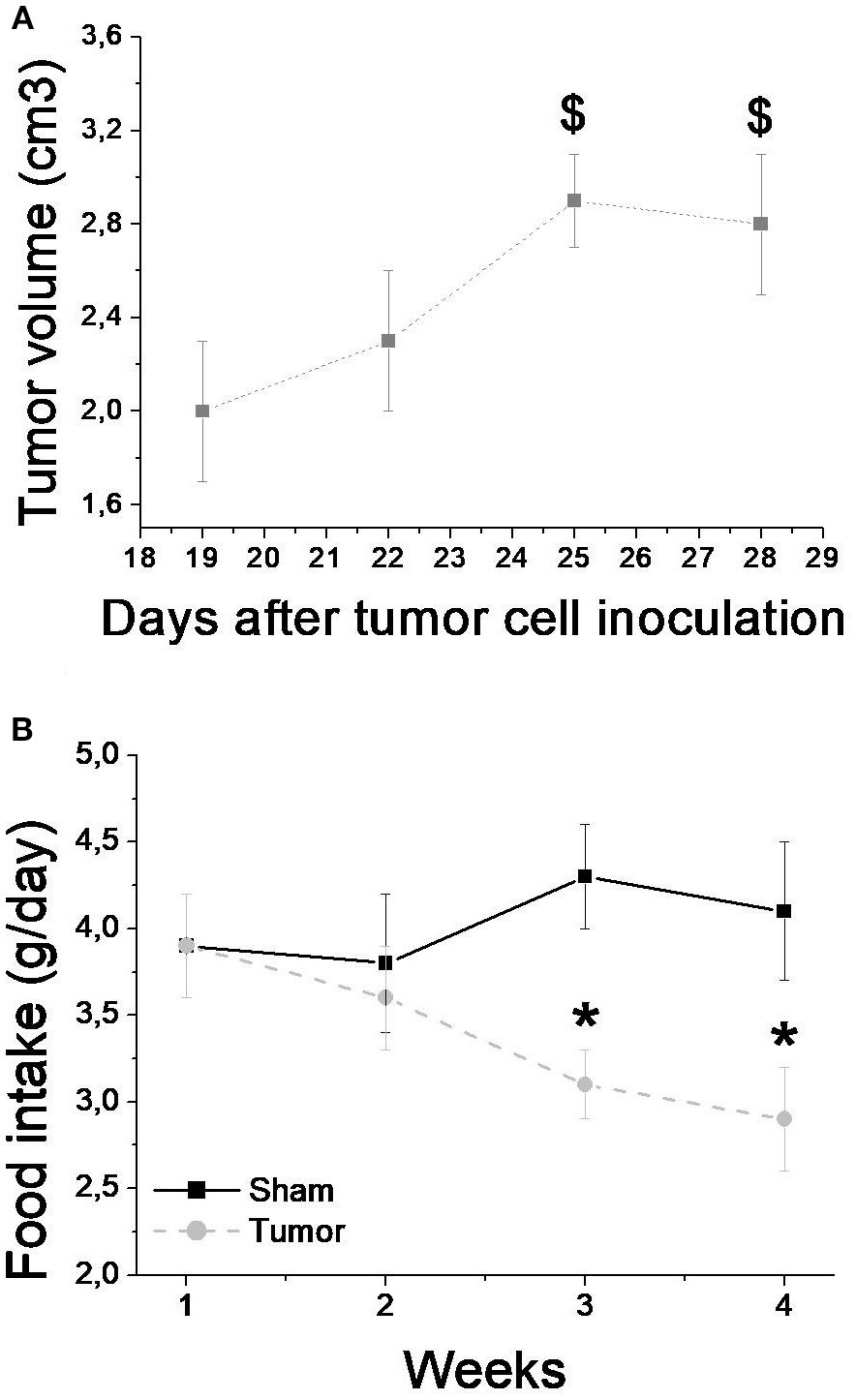
Tumor volume **(A)** and food intake **(B)** over the 4 weeks after tumor cell inoculation for sham (*n* = 8) and tumor (*n* = 8) groups. Values are mean ± SD. ^*^Indicates significant difference from S group; ^$^Indicate significant difference from week 1 (*P* < 0.05 by ANOVA 2-way for two independent samples).

Tumor mass was apparent only after 19 days after cell inoculation in average. The animals were monitored until euthanasia when the tumor mass reached 11.72% of total body weight. After careful analysis, we did not find any metastases in target tissues such as lung, stomach and intestines, extra-regional lymph nodes, liver, pancreas and spleen. Tumor development also caused decreased food intake over the third and fourth weeks (Figure 1), without changes in water intake.

The tissue responses of the tumor-bearing animals and the sham group are shown in Table 1. Clearly, the muscle and epididymal fat content were significantly decreased (*P* < 0.05) in tumor-bearing mice compared to the sham group. Spleen was 2-fold larger in tumor-bearing compared to Sham mice. Tumor-free body weight was not different, despite the *P* trend = 0.07 (Table 1). Cachexia index was 8.3%, higher than 5% established by as Fearon et al. (2011) as cachexia definition. Liver relative weight was not affected by tumor growth.

**Table 1:** Initial body mass, tumor-free body mass, relative weights of the liver, muscle, epididymal fat pads and tumor mass, as well as cachexia index determined for tumor-bearing and Sham mice.

	**Sham (*n* = 8)**	**Tumor (*n* = 8)**	***p*-value**
Initial body mass (g)	20.6 ± 0.8	20.0 ± 1.1	0.74
Tumor-free body mass (g)	21.2 ± 0.8	19.9 ± 1.91	0.07
Liver (%/body weight)	5.00 ± 0.72	5.25 ± 0.65	0.40
∑ muscle weigh (%/body weight)	0.59 ± 0.12	0.46 ± 0.11^*^	0.02
Spleen (%/body weight)	0.97 ± 0.11	2.23 ± 0.56	0.00
Epididymal fat (%/body weight)	1.18 ± 0.04	0.79 ± 0.03^*^	0.01
Tumor mass (%/body weight)	-	14.55 ± 9.92	-
Cachexia index (%)	-	8.34 ± 3.81	-

*Values expressed as mean ± SD. Data evaluated by T-test for independent samples; adopted value of significance of P < 0.05*.

**Figure 2:**
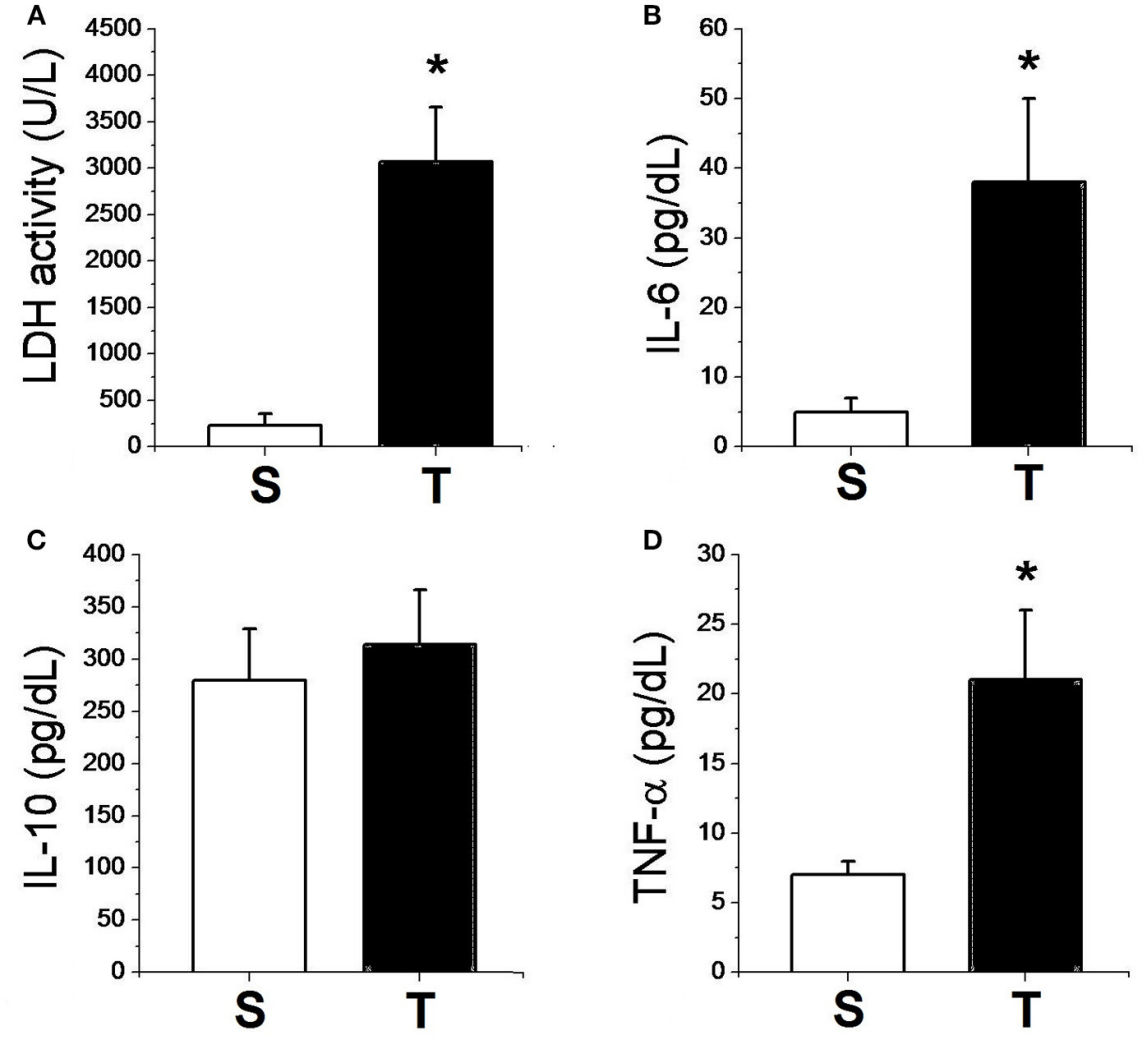
Plasma enzymatic activities of LDH **(A)** and plasma concentrations of IL-6 **(B)**, IL-10 **(C)**, TNF-α **(D)** for sham (*n* = 6) and tumor (*n* = 6) groups. Values are mean ± SD. ^*^Indicates significant difference from S group (*P* < 0.05 by *t*-test for independent samples).

Figure 2 shows the data related to the systemic response of experimental groups. The tumor-bearing mice presented elevated LDH plasma levels. Plasma inflammatory cytokines were significantly elevated in the tumor bearing group; TNF-α was over two-fold and IL-6 was over eight-fold higher in tumor-bearing mice compared to Sham animals. However, there was no difference between groups in relation to IL-10.

**Figure 3:**
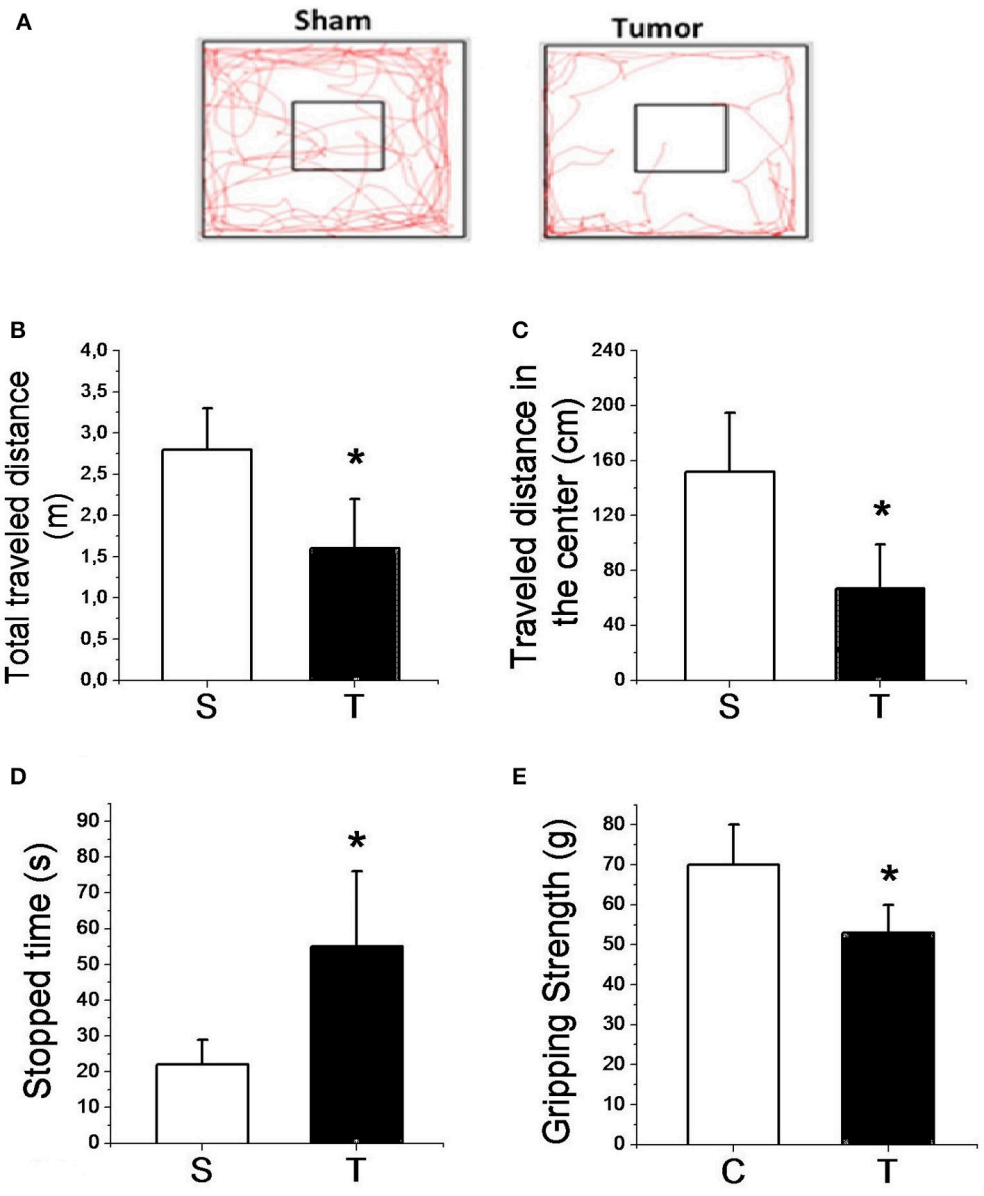
Representative figure of the lomocotion test **(A)**; total traveled distance **(B)**; traveled distance in center **(C)**; stopped time **(D)**; and muscle gripping strength **(E)** for sham (*n* = 8) and tumor (*n* = 8) groups. Values are mean ± SD. ^*^Indicates significant difference from S group (*P* < 0.05 by *t*-test for independent samples).

Figure 3 presents the results of the locomotor and exploratory test as well as the gripping strength test. The overall distance and distance traveled in the center were significantly (*P* < 0.05) lower (40 and 56%, respectively) in tumor-bearing mice compared to sham. Tumor-bearing mice also spent significantly (*P* < 0.05) more time stopped than sham mice. In addition, tumor growth promoted a significantly decreased muscle gripping strength compared to sham mice.

**Figure 4:**
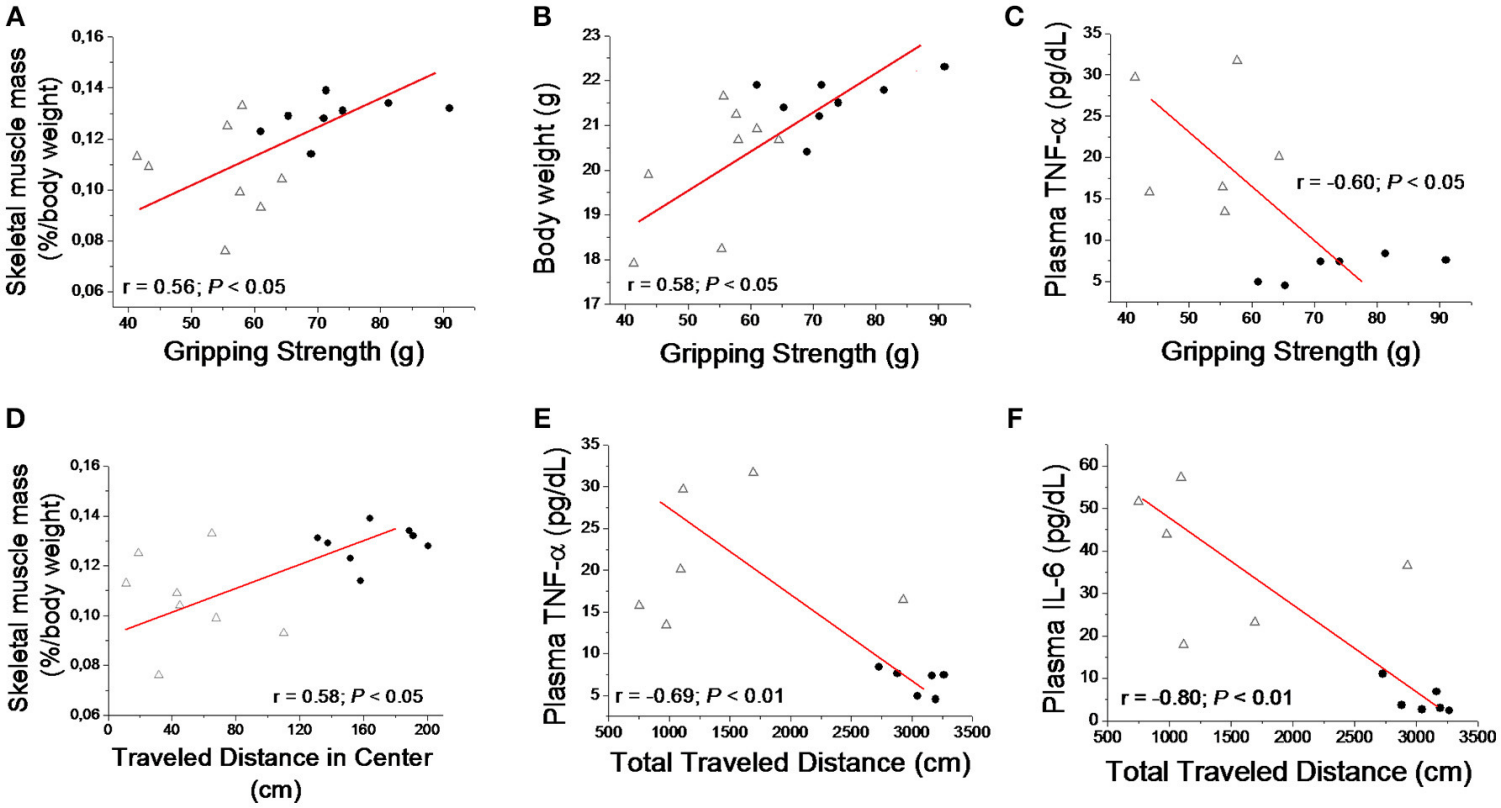
Correlation between muscle gripping strength with skeletal muscle mass **(A)**, total body weight **(B)** and plasma TNF-α **(C)**. Correlation was also determined between skeletal muscle mass and traveled distance in center at locomotor test **(D)**, and with total traveled distance at locomotor test with Plasma TNF- α **(E)** and IL-6 **(F)** for sham (•) and tumor (▴) groups.

The *Pearson* correlation test demonstrated reduced skeletal muscle mass and body weight loss were significantly (*P* < 0.05) associated to gripping strength. Elevated plasma TNF-α was inversely (*P* < 0.05) associated to gripping strength. Also, reduced skeletal muscle mass and elevated plasma TNF-α and IL-6 were determinant for the impaired locomotor activity parameters (Figure 4). In addition, correlation test also demonstrated negative association between decreased skeletal muscle mass and elevated plasma TNF-α (*r* = −0.71; *P* < 0.05) and IL-6 (*r* = −0.57; *P* < 0.05).

## Discussion

Our data demonstrated B16F10 tumor cell inoculation promoted cachexia characterized by weight loss, skeletal muscle wasting, adipose tissue loss and anorexia. Fat tissue significantly targeted presenting a 33.06% reduction in tumor-bearing mice compared to controls. The catabolic state appears to be intrinsic to the anorexia state and systemic organic pro-inflammatory response against tumor progressive growth. In addition, tumor growth-driven inflammation and muscle loss impaired voluntary muscle strength and decreased locomotor activity in tumor-bearing mice. Although previously studies have demonstrated B16F10 intraperitoneal inoculation was able to induce cancer cachexia (Kawamura et al., 1999a,b). Our data have demonstrated therefore, both muscle wasting and inflammatory condition mediated tumor growth play an important role in skeletal muscle strength loss and locomotor activity impairment.

Preclinical tumor models indicate that body and tissue wasting can impact functional parameters (Chen et al., 2015). Skeletal muscle loss appears to be the most essential component associated with poor outcome in cancer cachexia (Fearon et al., 2011). Our data highlighted reduced grip strength and decreased locomotor activity in the tumor group challenged with B16F10 cells, were associated to loss of muscle mass. These data demonstrated tumor growth promotes a critical modification in skeletal muscle that compromised animal's locomotor activity. Tumor-bearing mice inoculated with LLC cells demonstrated atrophy and lower grip strength (Chen et al., 2015). However, the skeletal muscle loss causative interaction is poorly investigated. Our results are particularly relevant since muscle loss has not been linked with lower muscle function only (Gabriel et al., 2006; Jorgensen et al., 2010). Due to complex coordination among neural, mechanic and physiological functions, muscle strength is highlighted as the most critical functional endpoint to survival and hospital discharge (Mendes et al., 2014; Joglekar et al., 2015).

Tumor-driven inflammatory factors may act as mechanisms underlying lipolysis and myolysis in wasting conditions (Der-Torossian et al., 2013; Laine et al., 2013; Inacio Pinto et al., 2015). Previously studies have demonstrated inflammatory cytokines play a critical role in muscle wasting by stimulating transcription factor nuclear factor-kB (NF-kB), which targets key skeletal muscular genes of catabolic cascade and up-regulate ubiquitin-proteasome pathway for protein catabolism (Zhou et al., 2016). In the subcutaneous tumor model, tumor-bearing mice show elevated TNF-α, IL-6 and activin A levels that up regulate ubiquitin ligases such as muscle ring finger-1 (MuRF1) and Atrogin-1 mRNA expression in gastrocnemius muscles (Matsuyama et al., 2015). Our data elucidates elevated plasma levels of TNF-α and IL-6 after 28 days of transplantation was associated to skeletal muscle loss, as demonstrated by negative association between decreased skeletal muscle mass and elevated plasma TNF-α (*r* = −0.71; *P* < 0.05) and IL-6 (*r* = −0.57; *P* < 0.05). Our study also demonstrated spleen was 2-fold larger in tumor-bearing compared to Sham mice which is a good indicator of systemic inflammation state. Indeed, chronic inflammatory condition caused by tumor development is thought to play a prominent role on muscle loss in cancer cachexia (De Larichaudy et al., 2012; Yuan et al., 2015). Although previous studies have suggested that IL-10 plays an important role in cachexia from different cancer types (Ebrahimi et al., 2004; Sun et al., 2010), our model promoted no changes in IL-10 plasma levels. In addition, taking the negative association between inflammatory interleukins and locomotor activity parameters (Figure 4), we may affirm that skeletal muscle wasting, loss of muscle strength and impaired locomotor activity imposed by B16F10 melanoma is inherent to the systemic pro-inflammatory condition promoted by tumor growth. Despite de association demonstrated among loss of muscle mass, elevated interleukin and impaired locomotion activity, is important to say that skeletal muscle tissue mass only is not the best predictor of muscle wasting. The absence of skeletal muscle cross sectional area and cardiac mass appears to be the principal limitation of the present study.

In addition to the catabolic-related inflammation scenario, a higher production of LDH is characteristic of a glycolytic phenotype reprogram. According to the Warburg effect, cancer cells undergo glycolysis rather than mitochondrial phosphorylation as a major source of energy (Vander Heiden et al., 2009), which may be directly linked with an activation of oncogenes and lower expression of tumor suppressors (Dang and Semenza, 1999; Allison et al., 2014). In melanoma patients, LDH is a stronger predictive factor of lower overall survival (Kelderman et al., 2014; Diem et al., 2015). However, the link between LDH and functional impairments in cancer is not clear. Indeed, melanoma cells favor a glycolytic phenotype and pro-inflammatory environment that may exert a key role in lipolysis, lower muscle strength and significant loss of mobility.

Anorexia is also an important component of cancer cachexia. Anorexia-related to cancer primary cause is often increased pro-inflammatory state and/or increased lactate production (Ezeoke and Morley, 2015). Administration of cytokines and lactate to rodents has been demonstrated to reduce food intake (Silberbauer et al., 2000; Patra and Arora, 2012). These two factors then modulate central nervous system neurotransmitter cascades, especially in hypothalamus, resulting in a reduction in satiation (Ezeoke and Morley, 2015). Therefore, is reasonable to affirm that elevated circulating interleukin and LDH activity demonstrated in our study may play a pivotal role in anorexia development induced by B16F10 melanoma development. Indeed, anorexia in cancer cachexia may also contribute significantly to muscle wasting and impaired locomotor activity demonstrated in our study. We cannot determine however, the significance plot of each cachexia component to locomotor activity impairment; it must be considered in future studies.

In conclusion, our data demonstrated that a subcutaneous B16F10 melanoma model promotes a catabolic state probably in response to a pro-inflammatory environment. Elevated inflammatory interleukins and muscle wasting induced by tumor development appears to play a important role on the impairment of muscle strength and decreased locomotor activity in tumor-bearing mice, some of the most critical endpoints associated with cancer morbidity and mortality. This model may be a valuable tool in testing new therapeutic approaches and future translational perspectives.

## Author contributions

FF and RD conceived of the studies. LV, GB, and FM provided essential equipment, expertise and resources. FF, CP, MT, PC, DR, and DdO performed experiments. FV, FF, and RD analyzed and interpreted data. FV, FF, and RD wrote and edited the manuscript.

### Conflict of interest statement

The authors declare that the research was conducted in the absence of any commercial or financial relationships that could be construed as a potential conflict of interest. The reviewer PT and handling editor declared their shared affiliation.
